# Cardiotoxicity of lung cancer-related immunotherapy versus chemotherapy: a systematic review and network meta-analysis of randomized controlled trials

**DOI:** 10.3389/fonc.2023.1158690

**Published:** 2023-04-14

**Authors:** Chengwei Jin, Jia Qi, Qilei Wang, Chenwei Pu, Mingming Tan

**Affiliations:** ^1^ Department of Cardiology, Zibo Central Hospital, Zibo, China; ^2^ Department of Respiratory and Critical Care Medicine, Zibo Central Hospital, Shandong, China

**Keywords:** lung cancer, cardiotoxicity, immunotherapy, CTLA-4, chemotherapy

## Abstract

**Background:**

Previous clinical randomized controlled trials (RCTs) have demonstrated that immune checkpoint inhibitors (ICIs) cause various toxicities during cancer treatment, but the effects of different inhibitors in combination with chemotherapy for cardiotoxicity remain controversial. The aim of the present study was to assess cardiotoxicity caused by programmed cell death protein 1 (PD-1), programmed cell death-Ligand 1 (PD-L1), and cytotoxic T lymphocyte associate protein-4 (CTLA-4) in combination with chemotherapy to treat lung cancer.

**Methods:**

The following ICIs were included in the present study: durvalumab, avelumab, ipilimumab, atezolizumab, pembrolizumab, cemiplimab, and nivolumab. The relevant information was extracted using a predefined data extraction table, and the risk of bias was assessed in randomized controlled trials using the Cochrane Bias Risk tool. The main outcomes were hypertension, heart failure, pericardial effusion, and other adverse cardiac events. The random effects model was used to conduct a paired meta-analysis, and a random effects network meta-analysis was then performed within a Bayesian framework.

**Results:**

In total, 17 RCTs were included in the present study. There were 11,063 individuals in the experimental and control groups, with an average age greater than 60 years. Based on the evaluation of all drug classes in RCTs, CTLA-4+chemotherapy (RR, -0.69 [95% CI, 2.91-1.52] and PD-L1 (RR, -0.21 [95% CI, -1.03-0.60]) were less cardiotoxic than the control arm, which indicated they were safer options for adverse cardiac events. PD-L1 alone was less cardiotoxic than PD-1 alone (RR, -0.57 [95% CI, -1.96-0.82]). Further, the dual immunotarget inhibitor, PD-1+CTLA-4, had the lowest SUCRA value and had the highest cardiotoxicity (SUCRA=9).

**Conclusion:**

When classified according to drug type, CTLA-4+chemotherapy is associated with fewer cardiac adverse events compared to other treatments. Dual immunotarget inhibitors are more likely to have adverse cardiac reactions. Therefore, clinicians should consider this evidence when developing an ICI immunotherapy regimen for lung cancer.

**Systematic review registration:**

https://www.crd.york.ac.uk/prospero, identifier CRD42023360931.

## Introduction

In the last five years, immune checkpoint inhibitors (ICIs) have become the first line of treatment for many types of cancer. Tumour cells express neoantigens, and immune cells recognize and destroy mutant proteins (1). Immune checkpoint inhibitors function by blocking inhibitory signals from tumour cells to T cells that recognize them, thus allowing tumour cells to be destroyed by the patient’s own immune system (2). Programmed death 1 (PD-1) is present on the surface of T cells and binds to programmed cell death 1 ligand 1 (PD-L1), which is widely expressed on tumour cells, allowing functional inhibition of the T cell response and tumour immune escape in several malignancies (3). Blocking the PD-1/PD-L1 interaction enhances immune recognition and stimulation of T cells to attack tumour cells. The ICIs that have been used in the clinic include cytotoxic T lymphocyte associate protein-4 (CTLA-4), PD-1, and PD-L1.

ICIs induce tumour responses in a variety of tumour types, including melanoma, non-small cell lung cancer (NSCLC), renal cell carcinoma (RCC), and Hodgkin’s disease. However, ICI treatment is often associated with immune-related adverse events and with multisystemic toxic changes that affect the skin, liver, nerves, and heart (4). ICI-induced cardiotoxicity is rare, with the incidence of specific types of cardiotoxicity as high as 1%, but it is usually serious and may be life-threatening (5). Patients may present with cardiac arrest, angina pectoris, myocarditis, cardiomyopathy, heart failure, and arrhythmia (6). In the present study, we discussed various immunotherapies known to cause cardiotoxicity. Understanding the interaction between lung cancer immunotherapy and the cardiac system will help in the early detection and prevention of cardiotoxicity. In this review, we focus on these differences and conduct qualitative assessments.

## Methods

### Search strategy

The meta-analysis was designed and conducted in accordance with the Preferred Reporting Project for Systematic Review and Meta-Analysis (PRISMA) reporting guidelines (7).

We conducted rigorous searches of the PubMed, Embase and Cochrane Library from inception to 1 December 2022 to identify all RCTs searched. The Medical Subject Heading (MeSH) terms retrieved and their entry terms were: “Immune Checkpoint Inhibitors”, “ICIs”, “PD-1 inhibitors”, “PD-L1 inhibitors”, “PD-1”, “PD-L1”, “CTLA-4”, “Durvalumab”, “Avelumab”, “Ipilimumab”, ”Atezolizumab”, ”Pembrolizumab”, “Cemiplimab”, “Nivolumab”, “Lung cancer”, “Cardiotoxicity” and “Adverse cardiac events”. The included articles were also limited to those published in English.

### Study selection and data extraction

The following medical records of lung cancer patients (>18 years) treated with ICIs were reviewed: all RCTs associated with PD-1/PD-L1 inhibitors or CTLA-4 inhibitors, including randomized controlled trials comparing PD-1, PD-L1 inhibitors, and CTLA-4 inhibitors with placebo or chemotherapy as well as those comparing PD-1, PD-L1 inhibitors, and CTLA-4 inhibitors with chemotherapy versus chemotherapy. Only studies that reported adverse cardiac events as well as those with extractable total number of trials and the number of events were included. Duplicate studies were excluded with literature management software. In addition, studies published as conference abstracts as well as case reports were excluded. Finally, trials that did not report treatment-related cardiotoxicity were also excluded.

Cardiotoxicity data were collected, including defined rates of heart disease and the number of individuals who experienced heart disease. Two researchers independently screened the titles and abstracts of publications, and any publication that the researchers deemed potentially relevant was evaluated in full-text. If disagreements occurred, they were resolved by discussion.

### Publication bias

Two researchers independently assessed the risk of bias of each included study according to the Cochrane Risk of Bias tool (8).

### Statistical analysis

The gemtc package in R (4.2.1) was used to perform the Bayesian network meta-analysis, and a random effects model was used for the network meta-analysis (9). The odds ratio (OR) and corresponding 95% confidence intervals (CI) were used as measures of comparison. Heterogeneity was assessed by the mtc.anohe command in the gemtc package. Heterogeneity between studies was assessed as high if I^2^> 50%, whereas heterogeneity between studies was considered to be low if I^2^< 50%. Trace plots and density plots were used to evaluate the convergence of the model. A P value < 0.05 (bilateral) was considered statistically significant (10). The hierarchy of treatments was determined by calculating ranking probabilities. In addition, for each outcome, the probability of each drug at each possible grade was estimated, and the probability distribution of each treatment for each possible position ranking was presented in the ranking graph. The surface under the cumulative ranking (SUCRA) curve was used to rank the safety of various immunotherapy regiments, in which lower SUCRA rankings indicate greater risks of cardiotoxicity (11). In addition to the network meta-analysis, Stata (15.1) tested publication bias and generated a funnel plot by running Egger’s command.

## Results

**Figure 1:**
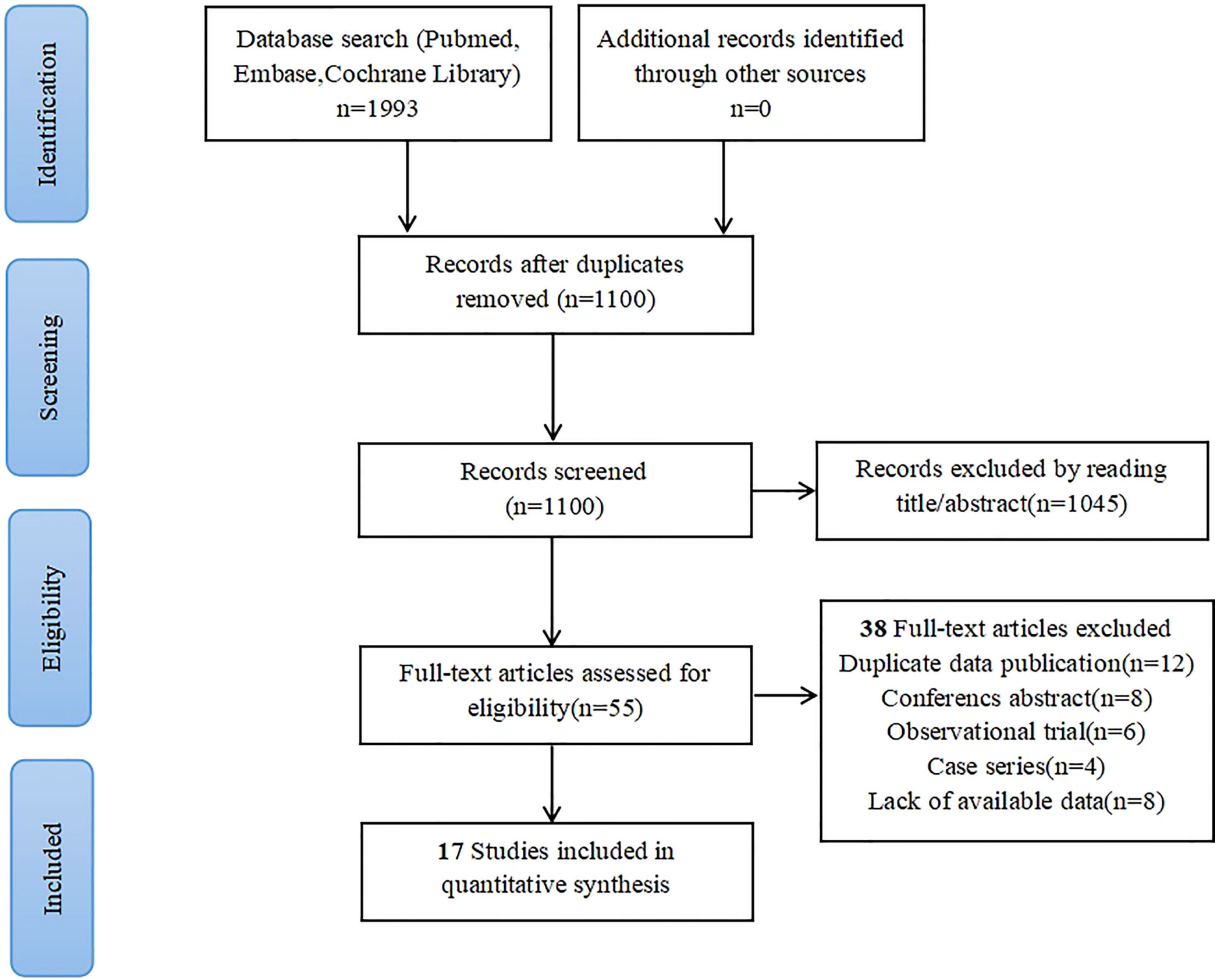
Flow chart of document screening.

**Figure 2:**
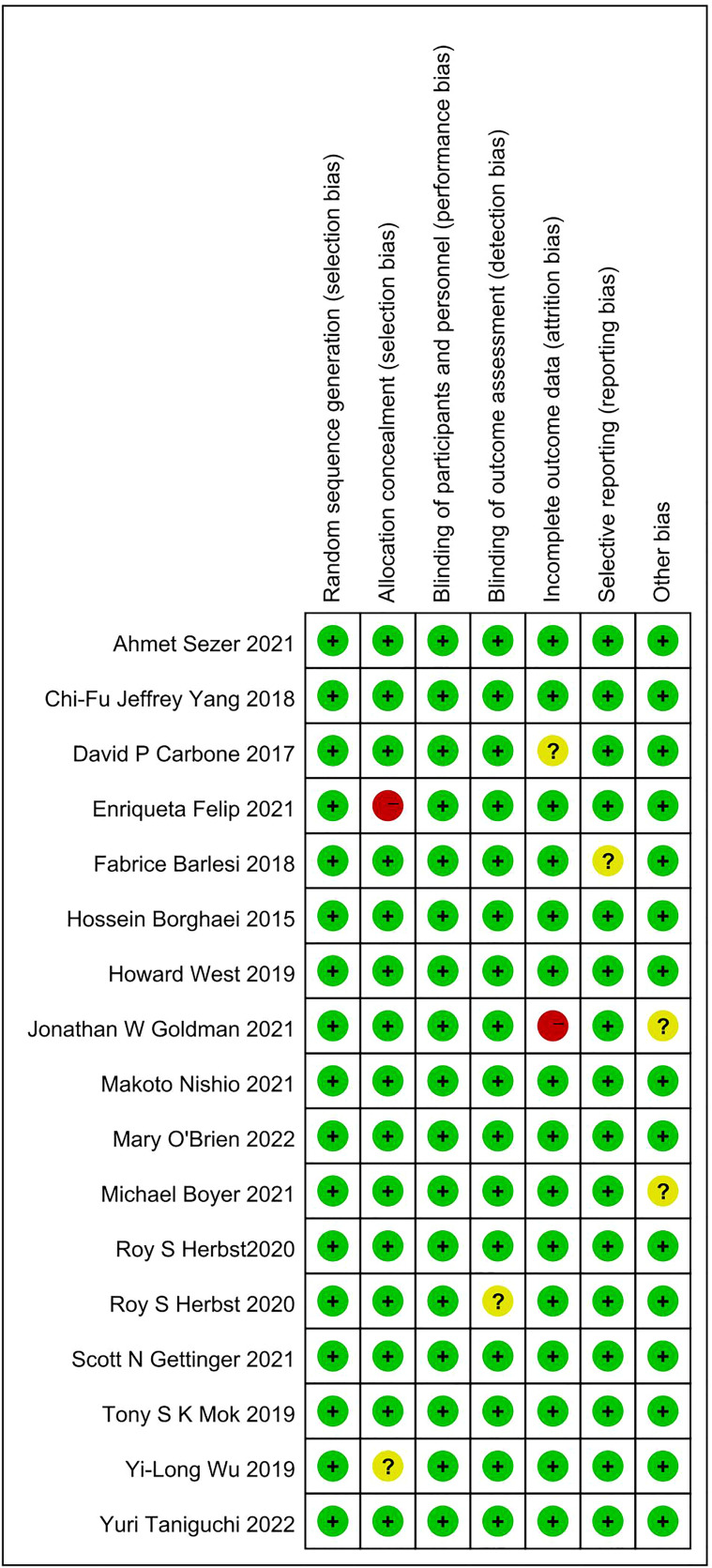
Risk of bias summary.

A search of the PubMed, Embase, and Cochrane libraries found 1100 potentially relevant records. After deleting duplicates and filtering titles and abstracts, 55 articles remained. After downloading and reading the full texts, 17 articles met the inclusion criteria and were included in the meta-analysis. One clinical trial had a high risk of bias, mainly due to incomplete report data and other biases (12). The flow chart of the search strategy and research selection is shown in **Figure 1**. In the meta-analysis, we used the Cochrane bias risk tool to evaluate the data quality. **Figure 2** shows a summary of the risk of bias in the included literature.

### Characteristics of clinical trials

**Figure 3:**
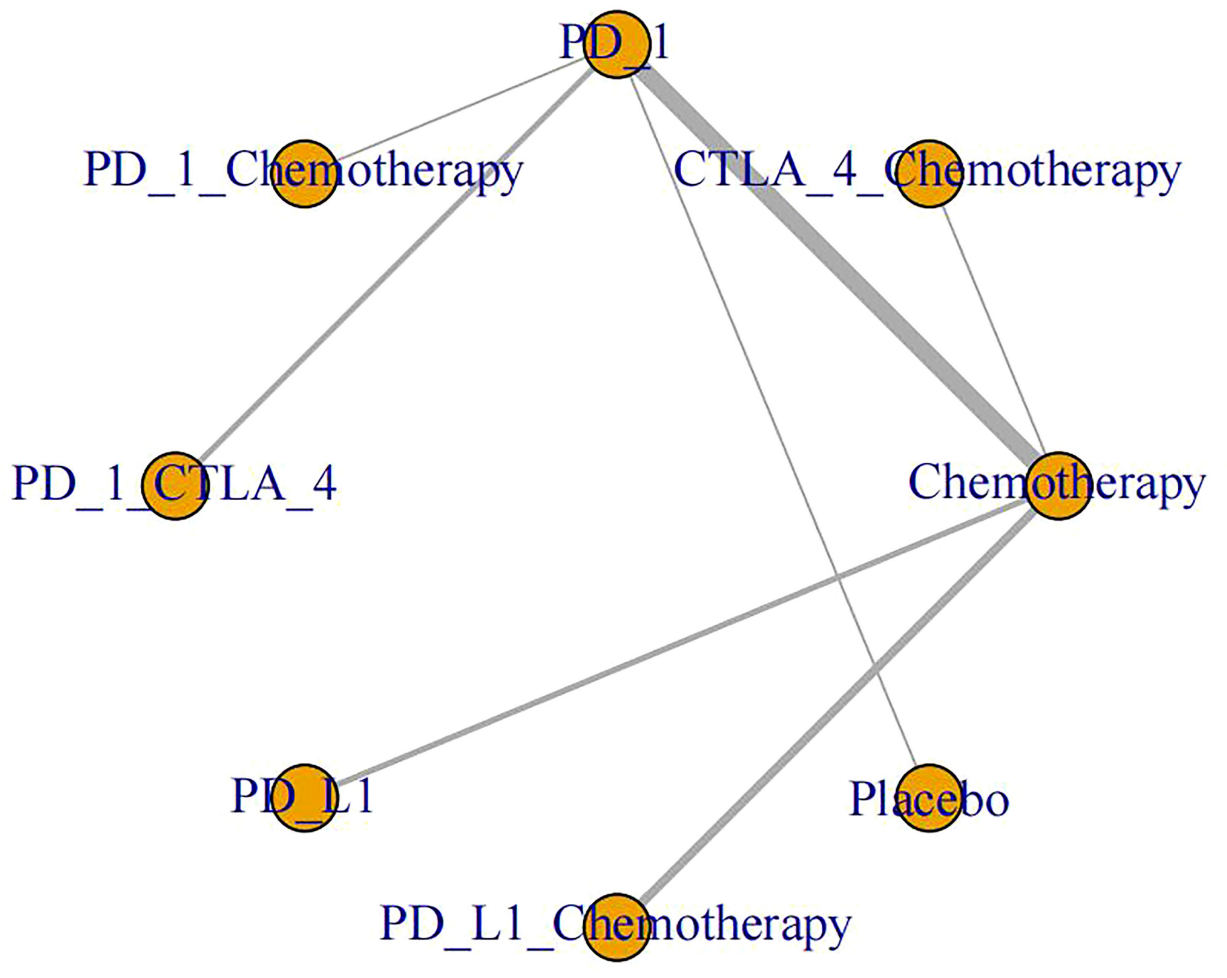
Network plots of different ICI comparisons. Single drug and drug combination comparison network. The size of each node is proportional to the number of participants (sample size). The lines represent the direct comparison available between treatment pairs, and the line width is proportional to the number of trials comparing each pair of treatments.

The specific experimental and control group characteristics of the 17 clinical trials are shown in **Table 1**. Seven ICIs were included, namely, durvalumab, avelumab, ipilimumab, atezolizumab, pembrolizumab, cemiplimab, and nivolumab. The following comparisons were made in the clinical studies: three studies compared PD-L1+chemotherapy to chemotherapy alone (13, 25, 26); two studies compared PD-L1 to chemotherapy (14, 24); two studies compared PD-1+CTLA-4 to PD-1 alone (27, 28); six studies compared PD-1 to chemotherapy (17, 19–23); one study compared PD-1 to placebo (15); one study compared PD-1+chemotherapy to PD-1 alone (16); and one study compared CTLA-4+chemotherapy to chemotherapy (12). **Figure 3** shows the network diagram of the comparisons of different immunosuppressant combinations. The average age of the participants in the 17 clinical trials was greater than 60 years old. **Table 2** shows the SUCRA ranking of the interventions based on treatment efficacy and cumulative probability plots, indicating the combination of immunological drugs that were least and most toxic to the heart in lung cancer patients. **Table 3** shows the specific rates of cardiotoxicity for different treatment combinations.

**Table 1:** Characteristics of included studies.

First Author	Year	Registration Number	Disease stage	PD-L1	Histology	Smoke	ECOG	Control armTreatment	Patients inControl arm (n)	Age	Experimentalarm treatment	Patientsin Experimentalarm (n)	Age	Medianfollow-up(month)
Jonathan W Goldman (13)	2021	NCT03043872	III 28 (10%) IV 240 (90%)	NA	NA	Never 15 (6%) Former 141 (53%) Current 112 (42%)	NA	platinum-etoposide	269	63 (57–68)	Durvalumab+tremelimumab + platinum-etoposide	268	63 (58–68)	25.1mo(IQR 22.3–27.9)
Fabrice Barlesi (14)	2018	NCT02395172	NA	Positive 264 (67%) Negative129 (33%)	Squamous 120(30%) Non-squamous 276 (70%)	Current or former 324 (82%) Never 70 (18%)	=0 144 (36%) =1 252 (64%)	Docetaxel	396	63 (57–69)	Avelumab	396	64 (58–69)	18.9mo(IQR 13.5-23.1)
Mary O'Brien (15)	2022	NCT02504372	IB 84 (14%) II 329 (56%) IIIA 177 (30%)	Positive 357 (60%) Negative 233 (39%)	Non-squamous 398 (67%) Squamous 192 (33%)	Current 75 (13%) Former 428 (73%) Never 87 (15%)	=0 380 (64%) =1 210 (36%)	Placebo	587	65.0 (59.0–70.0)	Pembrolizumab	590	65.0 (59.0–70.0)	35.6mo(IQR 27.1–45.5)
Chi-Fu Jeffrey Yang (12)	2018	NCT01820754	IIA 1 (8) IIB 2 (15) IIIA 10 (77)	NA	Adenocarcinoma 8 (62%) Squamous cell carcinoma 5 (38%)	Smoke 12 (92%) Non 1 (8%)	=0 13(100%)	Platinum-doublet Chemotherapy	42	62 (33–76)	Ipilimumab+Platinum-doublet Chemotherapy	13	59 (51–75)	NA
Yuri Taniguchi (16)	2022	jRCTs031180331	III 5 (7.8) IVA 29 (45.3) IVB 30 (46.9)	Positive 16 (24.9) Negative 12 (18.8)	Squamous 14 (21.9%) Nonsquamous 50 (78.1%)	Never 15 (23.4%) Ever 49 (76.6%)	NA	Nivolumab+ docetaxel	64	69 (45–83)	Nivolumab	64	69.5 (35–84)	18.9mo(IQR, 0.6-39.1)
Hossein Borghaei (17)	2015	NCT01673867	IIIB 20 (7) IV 272 (93)	NA	NA	Current/former 231 (79%) Never 58 (20%)	=0 84 (29) =1 208 (71)	Docetaxel	290	64 (21-85)	Nivolumab	292	61 (37-84)	Min:13.2mo
Enriqueta Felip (18)	2021	NCT02486718	IB 65 (13%) IIA 147 (29%) IIB 90 (18%) IIIA 205 (40%)	Positive 283 (56%) Negative 210 (41%)	Squamous179 (35%) Non-squamous 328 (65%)	Never 114 (23%) Previous 317 (63%) Current 65 (15%)	=0 273 (54%) =1 232 (46%) =2 2 (<1%)	Best Supportive Care	498	62 (56–68)	Atezolizumab	507	62 (57–67)	32.8mo (IQR 27.6–39.0)
Roy S Herbst (19)	2020	NCT01905657	NA	Positive 690 (100%)	Squamous 156 (22.6%) Nonsquamous 486 (70.4%)	Current or former 565 (81.9%) Never 123 (17.8)	=0 231 (33.5) =1 455 (65.9) =2 4 (0.6)	Docetaxel	343	64	Pembrolizumab	690	65	42.6mo(IQR, 35.2-53.2)
Tony S K Mok (20)	2019	NCT02220894	NA	Positive 637 (100%)	Squamous 114 (38%) Non-squamous 394 (62%)	Current 125 (20%) Former 370 (58%) Never 142 (22%)	=0 198(31%) =1 439(69%)	Platinum-based Chemotherap Y	637	63.0 (57.0-69.0)	Pembrolizumab	637	63.0 (57.0-69.0)	12.8mo(IQR 6.0-20.0)
Yi-Long Wu (21)	2019	NCT02613507	NA	Positive 168 (50) Negative 138 (41)	Squamous 133 (39%) Nonsquamous 205 (61%)	Current/former 236 (70%) Never 102 (30%)	=0 47 (14) =1 291 (86)	Docetaxel	166	60 (38 -78)	Nivolumab	388	60 (27 -78)	10.4mo(IQR: 0.2-21.1)
David P Carbone (22)	2017	NCT02041533	IV 255 (94) Recurrent 16 (6)	≥5% 208 (77) ≥50% 88 (32)	Squamous 66 (24%) Nonsquamous 205 (76%)	Never 30 (11%) Former 186 (69%) Current 52 (19%)	=0 85 (31) =1 183(68) ≥2 2 (1)	Platinum-based Chemotherapy	270	65(29-87)	Nivolumab	271	63(32-89)	13.5mo
Ahmet Sezer (23)	2021	NCT03088540	Locally advanced 63 (18%) Metastatic 293 (82%)	NA	Squamous 159 (45%) Non-squamous 197 (55%)	Current 133 (37%) Past 223 (63%)	=0 96 (27%) =1 260(73%)	Platinum-doublet Chemotherapy	354	64 (57–69)	Cemiplimab	356	63 (58–69)	10.8mo (IQR,7.6-15.8)
Roy S Herbst (24)	2020	NCT02409342	NA	NA	Nonsquamous 192 (69.3%) Squamous 85 (30.7%)	Never 37 (13.4%) Current 74 (26.7%) Previous 166 (59.9%)	=0 97 (35.0) =1 180 (65.0)	Platinum-based Chemotherapy	277	65 (30–87)	Atezolizumab	277	64 (30–81)	NA
Howard West (25)	2019	NCT02367781	NA	High 91 (19%) Low 139 (29%) negative253 (52%)	Adenocarcinoma 462 (96%) Adenosquamous 4 (1%)	Never 64 (13%) Current 96 (20%) Previous 323 (67%)	=0 204(42%) =1 278 (58%)	Carboplatin+Nab-paclitaxel	240	65 (38–85)	Atezolizumab+Carboplatin+Nab-paclitaxel	483	64 (18–86)	18.5mo(IQR,15.2–23.6)
Makoto Nishio (26)	2021	NA	NA	NA	Non-squamous 292(100%)	Never 37 (12.7%) Current or former 255 (87.3%)	=0 126 (43.2) =1 166 (56.8)	Carboplatin /cisplatin +pemetrexed	286	63.0 (33–83)	Atezolizumab+carboplatin/cisplatin +pemetrexed	292	64.0 (31–85)	28.4mo
Michael Boyer (27)	2021	NCT03302234	NA	NA	Squamous 77 (27.1%) Nonsquamous 207(72.9%)	Current 58 (20.4%) Former 197 (69.4%)	=0 101 (35.6) =1 183 (64.4)	Pembrolizumab+placebo	284	65(35-85)	Pembrolizumab+ipilimumab	284	64(35-85)	20.6 mo(IQR, 12.4-31.7)
Scott N Gettinger (28)	2021	NCT02785952	Stage IV	NA	Squamous Cell Lung Cancer	Current 48 (38%) Former 75 (60%)	NA	Nivolumab	127	68.1 (48.6-90.3)	Nivolumab+Ipilimumab	125	67.5 (41.8-83.4)	29.5mo(95%CI, 26.0-32.8)

ECOG, Eastern Cooperative Oncology Group; mo, month; IQR, Inter-Quartile Range; NA, not available.

**Table 2:** SUCRA ranking probabilities of different treatments.

Treatment	SUCRA	Rank
CTLA-4+Chemotherapy	80.1	1
PD-L1	75.2	2
Chemotherapy	67	3
Placebo	61.6	4
PD-1	46.4	5
PD-L1+Chemotherapy	34.2	6
PD-1+Chemotherapy	26.5	7
PD-1+CTLA-4	9	8

**Table 3:** The incidence of cardiotoxicity toxicity in different treatment groups.

Type of Cardiotoxicity	Myocarditis	Pericarditis	Arrhythmias	Myocardial infarction	Heartfailure	Cardiac arrest	Hypertension	Angina
**Chemo(%)**	0.06	NA	0.44	0.18	0.12	0.12	0.29	0.03
**PD-1(%)**	0.28	NA	0.11	0.08	0.03	NA	1.02	NA
**PD-L1(%)**	0.15	NA	NA	0.15	0.15	0.15	1.33	NA
**Placebo(%)**	0.17	NA	NA	0.69	NA	NA	5.51	NA
**PD-1+Chemo(%)**	1.56	NA	NA	NA	NA	NA	NA	NA
**PD-L1+Chemo(%)**	0.87	0.1	3.11	0.39	0.1	0.39	NA	0.19
**CTLA-4+Chemo(%)**	NA	NA	7.69	NA	NA	NA	NA	NA
**PD-1+CTLA-4(%)**	0.99	NA	NA	NA	0.25	0.25	0.99	NA

NA, not available.

### Risk of cardiotoxicity

**Figure 4:**
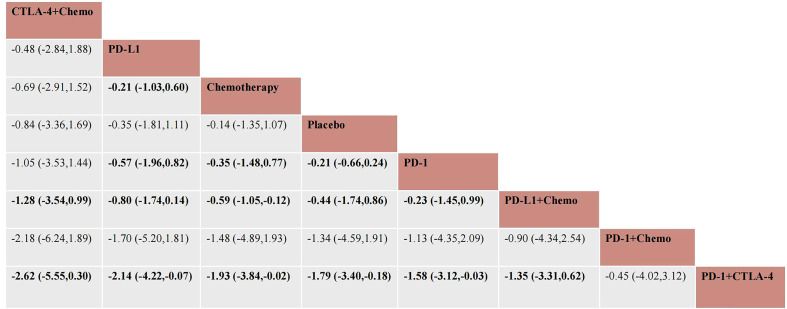
Network meta-analysis estimates for cardiotoxicity of different drug species. Ranking of drugs according to cardiac toxicity using SUCRA. The comparison should be read from left to right. Statistically significant differences are indicated in bold and underlined.

**Figure 5:**
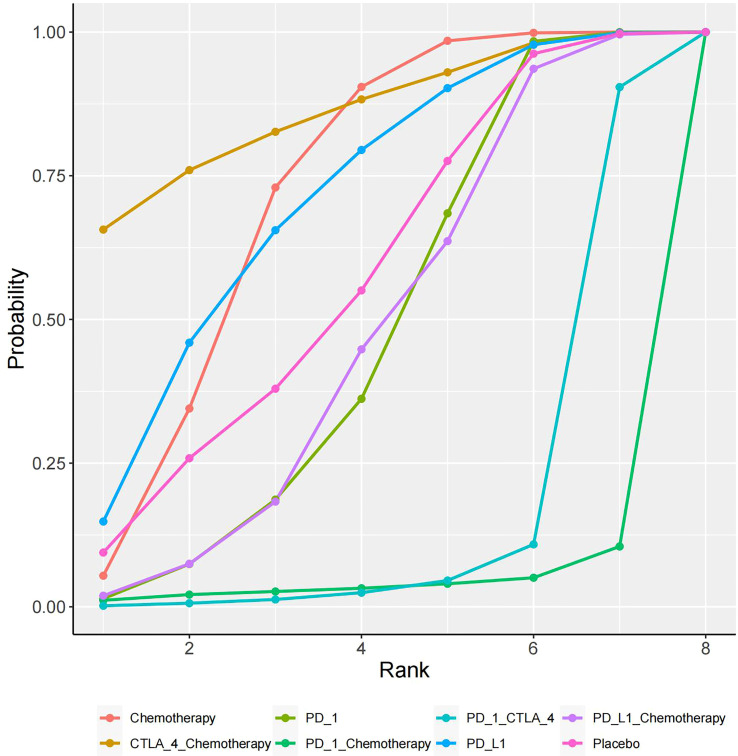
Cumulative ranking probability graph.

In total, 16 studies were included in a network meta-analysis of the cardiotoxic effects of single inhibitors and combinations in lung cancer. According to the SUCRA rankings, the top three interventions were CTLA-4+chemotherapy, PD-L1, and chemotherapy with SUCRA values of 80.1%, 75.2%, and 67%, respectively. The results of the network meta-analysis of the primary outcomes are shown in **Figure 4**. CTLA-4+chemotherapy had a lower rate of adverse cardiac events than PD-L1+chemotherapy (OR, -1.28 [95% CI, -3.54-0.99]) or placebo (OR, -0.84 [95% CI, -3.36-1.69]). The effect of PD-L1 alone on cardiac toxicity was significantly less than that of PD-1 alone (OR, -0.57 [95% CI, -1.96-0.82]). PD-1 alone was less cardiotoxic than the PD-1+CTLA-4 dual ICI (OR, -1.58 [95% CI, -3.12-0.18]) and PD-1+chemotherapy (OR, -1.13 [95% CI, -4.35-2.09]). The combination of PD-1 and CTLA-4 (SUCRA=9%), had the greatest cardiotoxicity and the highest probability of adverse cardiac events. **Figure 5** shows the cumulative ranking probability of each ICI combination.

**Figure 6:**
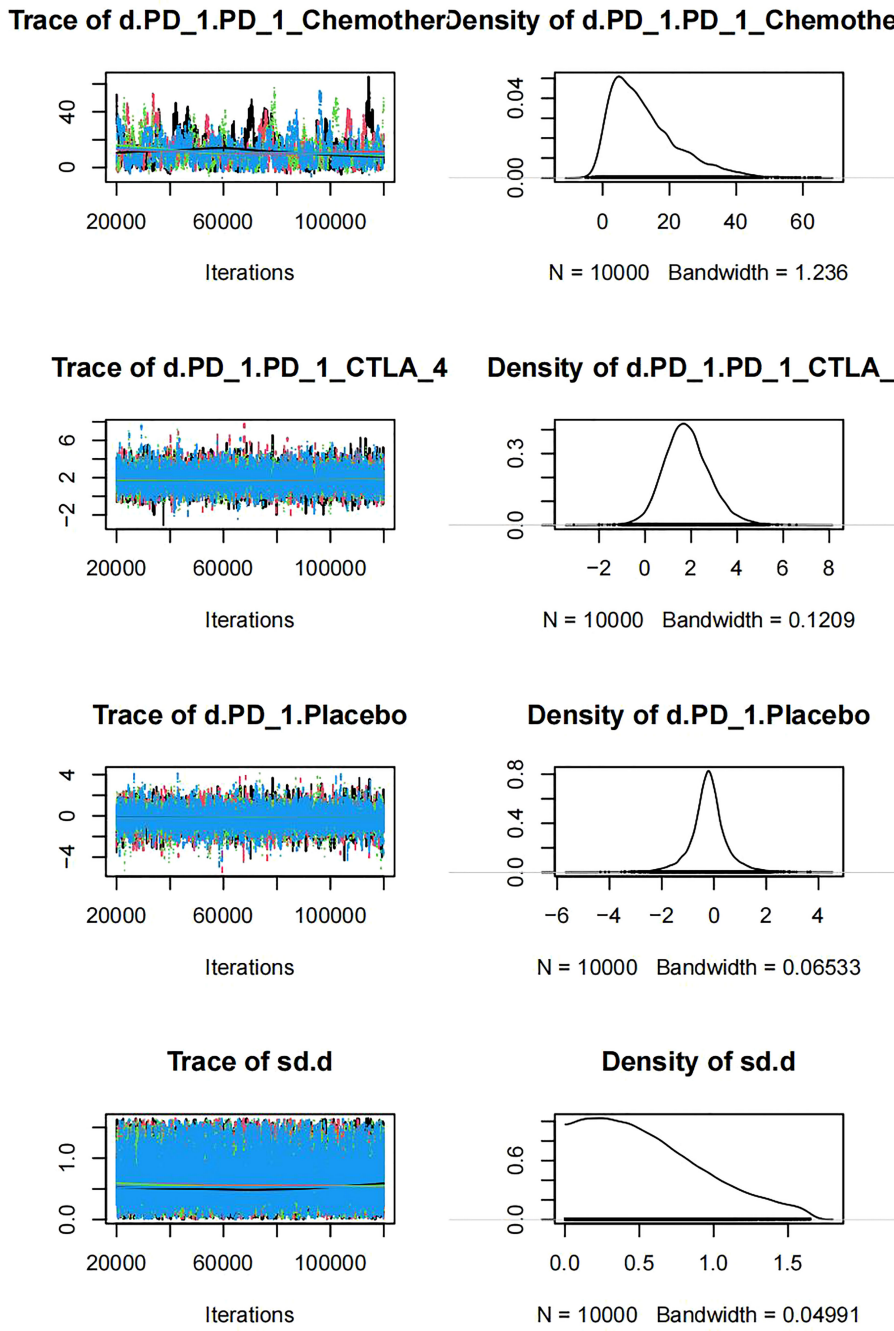
Track density map.

The trajectory indicated that when the number of iterations reached more than 5000, the MCMC chain reached a stable fusion from the initial portion, and the overlapping portion accounted for most of the chain fluctuation range in the subsequent calculation (**Figure 6**). The density graph indicated that when the number of iterations reached 20000, the bandwidth tended to be zero and reached stability, which indicated that the model converged well.

### Publication bias

**Figure 7:**
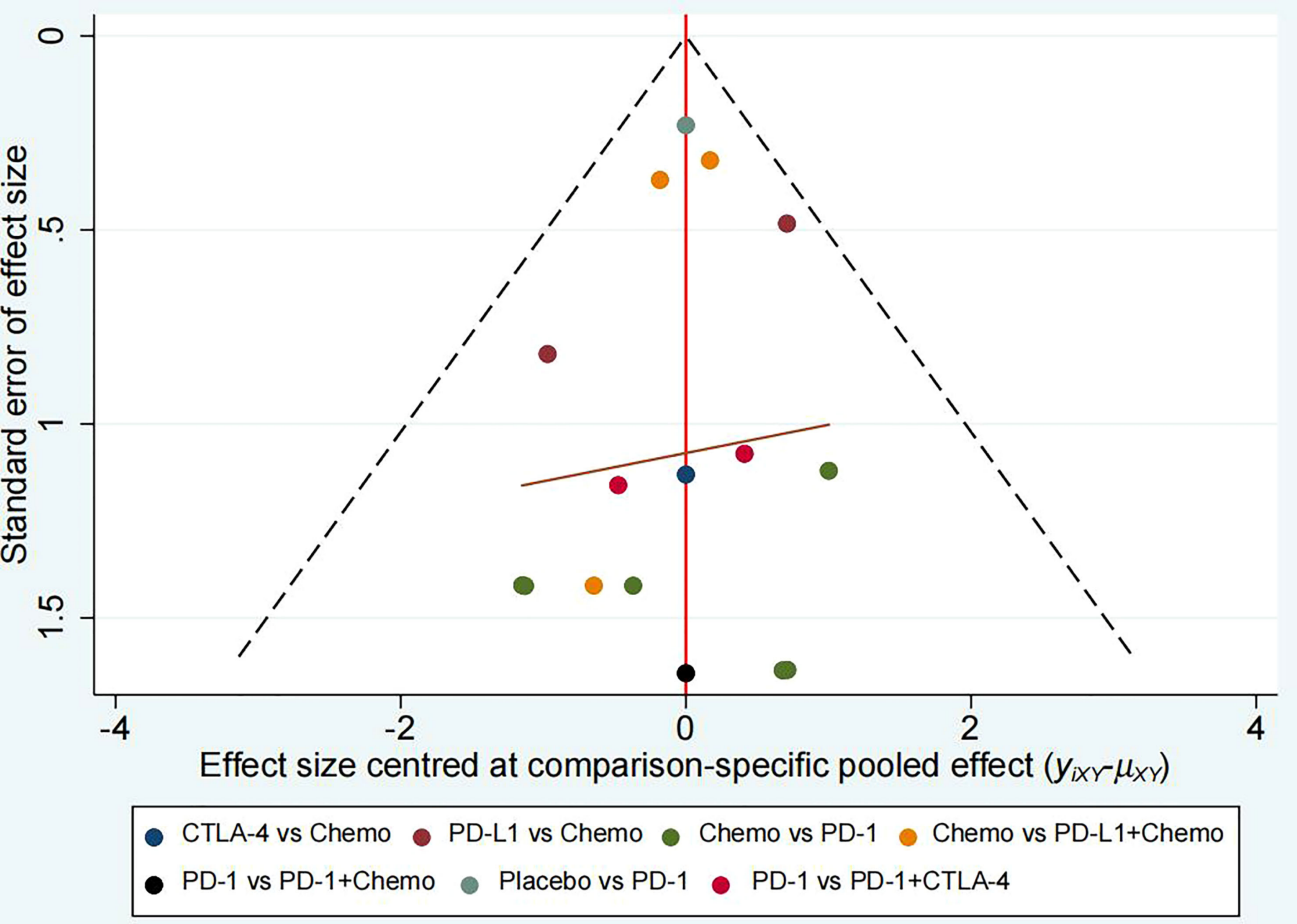
Comparison-adjusted funnel plot for mean overall change in cardiotoxicity in all comparisons.

There were no detectable differences in any of the other comparisons. The network analysis funnel plot was approximately symmetrical (**Figure 7**). The p value after Egger’s test was 0.949, which indicated that there was no significant publication bias in the data.

## Discussion

The present network meta-analysis evaluated cardiotoxicity following different combinations of immunosuppressants in lung cancer patients. The cumulative number of patients in the experimental arm of each included clinical trial was 5933.

The magnitude of cardiotoxic effect estimates varied widely across different combinations of immunosuppressants. The CTLA-4+chemotherapy and PD-L1 treatments were less toxic than placebo or control, while PD-L1 ranked higher than PD-1, which indicated less cardiotoxicity (SUCRA, 75.2% vs. 46.4%). The toxicity of PD-L1+chemotherapy (OR, -0.23 [95% CI, -1.45 to 0.99]) and PD-1+CTLA-4 (OR, -1.58 [95% CI, - 3.12–0.03]) and was greater than that of PD-1 treatment alone. Of note, the PD-1+chemotherapy treatment was not statistically significant and may lack clinical significance.

Among cancer survivors, cardiotoxicity is considered a major cause of long-term morbidity and mortality. When cancer patients benefit from anticancer drugs, they should also receive specific interventions to treat cardiotoxic complications (29). The Canadian Cardiovascular Society guidelines have recommended that ACE inhibitors or ARBs and/or β blockers and/or statins should be considered in patients at high risk of left ventricular dysfunction associated with cancer therapy to reduce the risk of cardiotoxicity (29). Clinically, captopril and enalapril have been used in cardioprotective strategies as chemotherapy adjuvants to reduce oxidative stress and minimize the production of free radicals to reduce cardiotoxicity (30).

ICIs have not been introduced for cancer treatment as checkpoint inhibitors until the last 5 years. Tumour cells have the ability to evade or quiesce the host immune system by utilizing immunomodulatory mechanisms (31). ICIs target these escape pathways, allowing the immune system to recognize and target cancer cells (32). ICI is a monoclonal antibody that targets the CTLA-4, PD-1, and PD-L1 inhibitory receptors. These drugs enable the immune system to attack tumour cells (32). Thus, mechanisms to prevent autoimmune responses are inhibited, explaining most of the potential autoimmune-related side effects associated with this class of drugs, such as rash, elevated aminotransferases, hypothyroidism, pneumonia, autoimmune hepatitis, and pituitary inflammation (33). The PD-1/PD-L1 pathway is essential for immune homeostasis within the myocardium and cardiac protection of T cells (34). It has been hypothesized that dysregulated immune cells falsely label surface structures, such as cardiolipin as antigens, resulting in subsequent targeting of normal cardiomyocytes or other cells expressing these antigens (35). The PD-1/PD-L1 pathway provides mechanisms of action for the various cardiotoxicities that occur after ICIs are used.

Myocarditis is inflammation of the heart muscle. ICI therapy can cause myocarditis with signs of dyspnoea, fatigue, and chest pain as well as elevated electrocardiogram (ECG) and myocardial enzyme profiles on examination (36). Similarly, ICIs can cause pericardial disorders, such as pericardial effusion and pericarditis (37). Cardiotoxicity is rare, occurring in only 0.04% to 1.14% of patients receiving immunotherapy (38). The present study indicated that receiving dual ICI combination therapy (e.g., CTLA-4 inhibitor combined with PD-1 inhibitor) was the clearest risk factor for ICI-related cardiotoxicity. Previous studies have reported that the combination of nivolumab and ipilimumab is associated with a 4.74-fold risk of myocarditis compared to nivolumab alone. Compared to myocarditis caused by ICI monotherapy, myocarditis caused by ICI combination therapy is also more likely to be severe (39). A previous trial of patients with advanced renal cell carcinoma who received avelumab and axitinib (antiangiogenic therapy) has indicated that the incidence of fatal myocarditis is 2% (40). Experimental models using transgenic mice have shown that immune checkpoints play a key role in the heart muscle; inflammation is especially harmful in this case because the heart muscle lacks redundancy and cannot regenerate (41). The integrity of PD-1, PD-L1, and CTLA-4 signalling is critical for downregulating excessive immune responses in the myocardia. Dilated cardiomyopathy and premature death in PD-1-deficient mice are due to a high titre of IgG autoantibodies against cardiac troponin I, which increases voltage-dependent L-type calcium currents in normal cardiomyocytes (42).

Drugs may have controllable safety as a single drug treatment, but combined use may induce severe AEs in susceptible patients. Among various types of cancer, tumours with high tumour mutational burden (TMB) caused by dysfunction during the DNA damage response (DDR) may have better clinical outcomes when receiving ICI treatment, such as CTLA-4 and PD-1 (43). The combination of PD-1 and CTLA-4 blockers is associated with an increased incidence of adverse events, accompanied by severe myocarditis in some patients (44). Bintrafusp alfa, a bifunctional fusion protein consisting of the transforming growth factor β (TGF-β) receptor fused to a human immunoglobulin G1 antibody that blocks programmed death ligand 1 (PD-L1), has been evaluated for safety and efficacy in patients with advanced NSCLC, breast cancer, and pancreatic cancer (45–47). Mitra et al. administered TGF-β in combination with PD-L1 antibody to mice for 5 weeks and observed acute bleeding and cardiovascular toxicity (48). In a total of 31 patients with recurrent gastric cancer treated with Bintrafusp alfa, Kang et al. reported that 6 patients (19%) experienced grade 3 treatment-related adverse events and no grade 4 events with a disease control rate of 26% (49). In addition, Kang et al. reported that 59 patients with advanced HPV-related cancer were treated with Bintrafusp alfa and had a clinical response rate of 35.6%. Moreover, treatment-related adverse events occurred in 49 patients (83.1%), and grade 3 or 4 adverse events occurred in 16 patients (27.1%). No treatment-related deaths occurred during follow-up (50). The median overall survival of patients with biliary tract cancer was 12.7 months, and the probability of grade 3 or above TRAE was 37% (51).

In patients treated with ICIs, various types of arrhythmias have been reported, including life-threatening complete atrioventricular block or ventricular tachyarrhythmia. The most common is atrial fibrillation followed by ventricular tachycardia or ventricular fibrillation and atrioventricular conduction disorder. Conduction disorders are associated with increased cardiovascular mortality in patients treated with ICIs (52). The pathological mechanism of arrhythmia induced by immunotherapy remains unclear. Once an ECG shows a prolonged PR interval, QT interval, and bundle branch block, the threshold for introducing cardiac pacing should be lowered because the conduction disorder may rapidly progress to late cardiac block (53). To avoid fatal adverse events, all patients receiving ICI treatment should undergo regular ECG screening.

Management Principles The treatment strategies for ICI-related cardiovascular complications were multifold as follows: discontinuation or reduction of ICI to prevent further toxicity; immunosuppression to alleviate inflammatory changes; and supportive therapy to address cardiac complications. Due to the complexity involved and the limited data available, the management of cardiotoxicity with ICI treatment should be conducted through discussions between oncologists and cardiologists. Patients should be given continuous ECG and haemodynamic monitoring when severe adverse cardiac events occur (54, 55). Immunosuppressive therapy for ICI-associated myocarditis requires large doses of corticosteroids. In cases that do not respond to corticosteroids, both recommendations suggest considering the use of infliximab (54, 55). It should be noted that infliximab may induce exacerbation of severe heart failure. Elevated serum troponin T levels have been used to assess the prognosis and diagnosis of major cardiovascular adverse events. In addition to the use of troponin, natriuretic peptide has also been proposed for screening and surveillance in high-risk patients with immune checkpoint inhibitor-associated myocarditis (38). Patients using ICIs still need regular monitoring of cardiac function in the clinic, such as cardiac troponin, ECG, and cardiac ultrasound. Genetic and tumour-specific factors of the patient also need to be considered when selecting immunotherapy and combination regimens to avoid resistance and adverse effects to these therapies (56). Clinicians should keep a close eye on the clinical manifestations during immunotherapy.

The present study had several limitations. First, because data from clinical trials were analysed, confounding factors, such as the underlying disease and previous treatment of the patient, cannot be ruled out. Second, due to data limitations, no group histological analysis was performed for lung squamous cell carcinoma and adenocarcinoma. Third, the control arm had different chemotherapy drugs and combinations as well as different dose regimens and different study durations included in the trial, which may lead to data bias. As there were few trials, we were unable to divide them in more detail.

## Conclusion

The present meta-analysis compared cardiac adverse events following ICI therapy in lung cancer patients. The network meta-analysis showed that CTLA-4+chemotherapy and PD-L1 are least likely to cause cardiotoxicity when classified by inhibitor combination. Moreover, PD-1 alone is more cardiotoxic than PD-L1, and treatment with the PD-1+CTLA-4 dual ICI caused the highest risk of adverse cardiac events. Therefore, evidence of cardiotoxicity should be considered when assessing the benefits and risks of ICI in the treatment of lung cancer.

## Data availability statement

The original contributions presented in the study are included in the article/supplementary material. Further inquiries can be directed to the corresponding author.

## Author contributions

Conceptualization: CJ, MT. Data curation: CJ, JQ, QW. Formal analysis: CP, CJ. Investigation: MT. Project administration: CJ, MT, JQ. Software: JQ, QW. Supervision: MT. Writing—original draft: JQ, QW, CP. Writing—review, and editing: CJ. All authors read and approved the final manuscript. All authors contributed to the article and approved the submitted version.
